# Co-designing discharge communication interventions for mental health visits to the pediatric emergency department: a mixed-methods study

**DOI:** 10.1186/s40900-024-00594-y

**Published:** 2024-06-21

**Authors:** Amber Z. Ali, Bruce Wright, Janet A. Curran, Joelle Fawcett-Arsenault, Amanda S. Newton

**Affiliations:** 1 Department of Pediatrics, Faculty of Medicine and Dentistry, University of Alberta, Edmonton Clinic Health Academy, 11405-87 Avenue, Edmonton, AB T6G 1C9 Canada; 2 School of Nursing, Dalhousie University, Halifax, NS Canada; 3 Alberta Health Services, Edmonton, AB Canada; 4 Women and Children’s Health Research Institute, Department of Pediatrics, Faculty of Medicine and Dentistry, University of Alberta, Edmonton, Canada

**Keywords:** Mental disorders, Discharge communication, Co-design, Patient and public involvement, Pediatric emergency medicine

## Abstract

**Background:**

Discharge communication is essential to convey information regarding the care provided and follow-up plans after a visit to a hospital emergency department (ED), but it can be lacking for visits for pediatric mental health crises. Our objective was to co-design and conduct usability testing of new discharge communication interventions to improve pediatric mental health discharge communication.

**Methods:**

The study was conducted in two phases using experience-based co-design (EBCD). In phase 1 (Sep 2021 to Jan 2022), five meetings were conducted with a team of six parents and two clinicians to co-design new ED discharge communication interventions for pediatric mental health care. Thematic analysis was used to identify patterns in team discussions and participant feedback related to discharge communication improvement and the Capability, Opportunity, Motivation, Behavior (COM-B) model was used to identify strategies to support the delivery of the new interventions. After meeting five, team members completed the Public and Patient Engagement Evaluation Tool (PPEET) to evaluate the co-design experience. In phase 2 (Apr to Jul 2022), intervention usability and satisfaction were evaluated by a new group of parents, youth aged 16–24 years, ED physicians, and nurses (*n* = 2 of each). Thematic analysis was used to identify usability issues and a validated 5-point Likert survey was used to evaluate user satisfaction. Evaluation results were used by the co-design team to finalize the interventions and delivery strategies.

**Results:**

Two discharge communication interventions were created: a brochure for families and clinicians to use during the ED visit, and a text-messaging system for families after the visit. There was high satisfaction with engagement in phase 1 (overall mean PPEET score, 4.5/5). In phase 2, user satisfaction was high (mean clinician score, 4.4/5; mean caregiver/youth score, 4.1/5) with both interventions. Usability feedback included in the final intervention versions included instructions on intervention use and ensuring the text-messaging system activates within 12–24 h of discharge.

**Conclusions:**

The interventions produced by this co-design initiative have the potential to address gaps in current discharge practices. Future testing is required to evaluate the impact on patients, caregivers, and health care system use after the ED visit.

**Supplementary Information:**

The online version contains supplementary material available at 10.1186/s40900-024-00594-y.

## Abstract (plain language summary)

Discharge communication is an important component of an emergency department (ED) visit for a mental health crisis as most children who visit the ED for mental health care are discharged home. To date, patients and their caregivers have not been involved in developing discharge communication interventions for this type of care. Our aim was to involve patients and caregivers to improve the communication provided to children and their caregivers during ED visits for mental health crises. We established a design team made up of six parents and two clinicians to design two new discharge communication interventions: a brochure for families and clinicians to use together during the ED visit, and a text-messaging system to support families after the visit. We tested how useable these interventions were with four other ED health care providers, two parents, and two youth. These participants reported high user satisfaction with the brochure, and usability feedback was used by the design team to improve the final versions of the two interventions. At the end of the project, the design team reported high satisfaction with their engagement experiences with the project. The interventions created by the team have the potential to address knowns gaps in current discharge practices, but future testing is required to evaluate the impact of these interventions on patients, caregivers, and health care system use after the ED visit.

**Supplementary Information:**

The online version contains supplementary material available at 10.1186/s40900-024-00594-y.

## Background

Emergency department (ED) health care providers have an integral role in mental health assessment, acute mental health care, and referral to specialized services [1–3]. There is, however, considerable variation across these clinical practices owing to a lack of policy and guidelines to standardize practices [4]. This includes most EDs not requiring the use of pediatric-specific tools to guide mental health assessments or having patient-centred procedures in place for care and referral practices [5–7]. This clinical context can result in ED health care providers feeling inadequately trained, unprepared, and uncomfortable in providing mental health care [2, 8].

Most children and adolescents who visit the ED for a mental health crisis will be discharged home [4, 9, 10], making discharge communication a critical component of the ED visit [10, 11]. Before leaving the ED, pediatric patients and their parents/caregivers should understand findings from mental health assessments, the ED care provided, and know if follow-up recommendations include the need (and reason) for specialized services. Parents/caregivers have also reported wanting information on how to help their child deal with the next crisis and how to support themselves [8]. Past research has indicated, however, that 32–48% of families do not receive any discharge instructions [4], and if instructions are provided, they are often briefly explained with crucial details missing [12], and may not be well understood by patients or parents/caregivers [8, 11].

Despite the significant role that parents/caregivers, pediatric patients, and health care providers play in the discharge communication process, they have not been involved in developing discharge interventions [12]. The involvement of children/adolescents and parents/caregivers in the development of discharge inventions for mental health care could improve access to treatment and services after the ED visit and increase the quality and appropriateness of discharge interventions provided in the ED [13, 14]. Patient engagement is a term used to describe a meaningful and active partnership between clinicians, researchers, and patients when conducting research, setting priorities, and translating study findings [15]. The term ‘patient’ typically includes people with health conditions, their caregivers, and/or family members [16]. In recent years, there is a growing consensus about the vital role of engaging patients in research [17]. Research suggests that patient engagement can help to improve the efficiency, effectiveness, and quality of health care services [13]. To date, most engagement initiatives have been limited to engaging either patients and parents/caregivers, or health care providers, rather than both [18]. To overcome this oversight in traditional patient engagement methods, experience-based co-design (EBCD) is being utilized [18].

EBCD is a qualitative framework, which can be used to evaluate and improve health care services through direct patient engagement [19]. The framework supports patients and clinicians collaborating to co-design health care improvement initiatives [20], and consists of 6 stages: (1) project set-up and observations, (2) engage staff and gather experiences, (3) engage patients/caregivers and gather experiences, (4) joint co-design event, (5) design and implement solutions, and (6) celebration event and review service improvements [21, 22]. At this time, the EBCD framework has been largely used in medical settings; there are limited published studies using EBCD in mental health care settings [19].

The aim of this study was to co-design new discharge communication interventions using the EBCD framework and test the usability of these interventions to improve pediatric mental health discharge communication in the ED.

## Methods

### Design

The study was mixed-method in design and conducted in two phases [12]. In phase 1, a team of parents/caregivers and ED health care providers co-designed the discharge communication interventions. This phase was based on the EBCD framework to ensure the quality and appropriateness of the interventions [13, 14, 23]. In phase 2, a group of youth aged 16–24 years, parents/caregivers, and ED health care providers evaluated the usability of the interventions with feedback used by the co-design team to finalize them.

The study was based out of the Stollery Children’s Hospital ED in Edmonton, Alberta, Canada, which has approximately 1800 annual visits by children aged 5–16 years for mental health concerns. The study was approved by the University of Alberta Research Ethics Board. The Guidance for Reporting Involvement of Patients and the Public—GRIPP2 checklist was used to report the findings of this co-design study (see Additional file 1) [24].

### Participants

Recruitment for both phases involved purposeful sampling to include participants with the experiences and expertise necessary for the study [25]. All participants provided informed consent. The time and lived experiences of parents/caregivers and youth were recognized by providing them with gift cards of their choice (phase 1 participants: $50 CAD per meeting; phase 2 participants: $25 CAD). Table 1 presents the demographics for individuals involved in both phases. In phase 1, demographics were collected from participants using the Public and Patient Engagement Evaluation Tool (PPEET), a tool developed to assess the quality and impact of engagement activities [26, 27]. In phase 2, demographics were collected as part of the user satisfaction survey [28].

**Table 1:** Characteristics of phase 1 and phase 2 participants, n (%)

Characteristic	Phase 1 (*n* = 8)	Phase 2 (*n* = 8)
**Gender**		
Male	2 (25.0)	2 (25.0)
Female	6 (75.0)	6 (75.0)
**Age, years**		
16–24	1 (12.5)	2 (25.0)
25–35		2 (25.0)
36–45	4 (50.0)	
46–55	3 (37.5)	2 (25.0)
56–65		2 (25.0)
**Perspective brought to the project**		
Family member/caregiver	6 (75.0)	2 (25.0)
Youth		2 (25.0)
Health care provider	2 (25.0)	4 (50.0)
**Group membership**		
Visible minority	1 (12.5)	3 (37.5)
LGBTQ community	1 (12.5)	
Person with disabilities		
Indigenous peoples of Canada		
Recent immigrant to Canada		
Not a member of one these groups	6 (75.0)	5 (62.5)
**Education**		
High school diploma		1 (25.0)
Some post-secondary training (college, university, technical)	1 (12.5)	
Completed college	2 (25.0)	1 (25.0)
Completed university	1 (12.5)	1 (25.0)
Post-graduate profession or graduate degree	4 (50.0)	5 (25.0)
**Worked for pay in a health care profession**		
Yes	4 (50.0)	
No	4 (50.0)	

LGBTQ: Lesbian, Gay, Bisexual, Transgender, Queer

In phase 1, the target size of the team was ~ 7 participants, in keeping with other mental health intervention co-design studies [29–31]. We recruited 6 parent/caregiver participants with lived experience in pediatric ED mental health visits through the Stollery Patient and Family-Centered Care Team, and two ED health care providers (one nurse, one physician) with experience in providing care for pediatric mental health concerns through a staff listserv email and staff meetings. We wanted to recruit one or two adolescents/youth with lived experience, but none expressed interest in participation.

The target sample size for phase 2 was ~ 8 participants per usability testing round [32–34]. While other usability studies have reported that three to four participants are adequate to find 80% of design usability problems [35], we wanted to include adolescents/youth, parents/caregivers, and health care providers in testing. We recruited participants for one round of testing. Although multiple testing rounds can be used to improve intervention usability, we only needed to conduct one round given the nominal usability issues that were identified. Usability participants were two youth and two parents/caregivers, all with lived experience, who were recruited through emails sent to the Stollery Youth Advisory Council and posters in the ED waiting room. ED health care providers were two physicians and two mental health nurses who did not participate in phase 1, but expressed interest in the study.

### Phase 1 methods

We developed the discharge communication interventions over five, virtually held, co-design team meetings. Each meeting lasted approximately 60 to 90 minutes. Meetings were co-led by two research team members with input from the patient and family-centred care coordinator from the hospital (author JFA). Meetings were recorded to facilitate data analysis and recordings were utilized to create a log that included attendance, length of meetings, discussion related to intervention design and discharge communication features (touchpoints, improvement targets, etc.), and the presence of decisional conflicts.

**Fig. 1:**
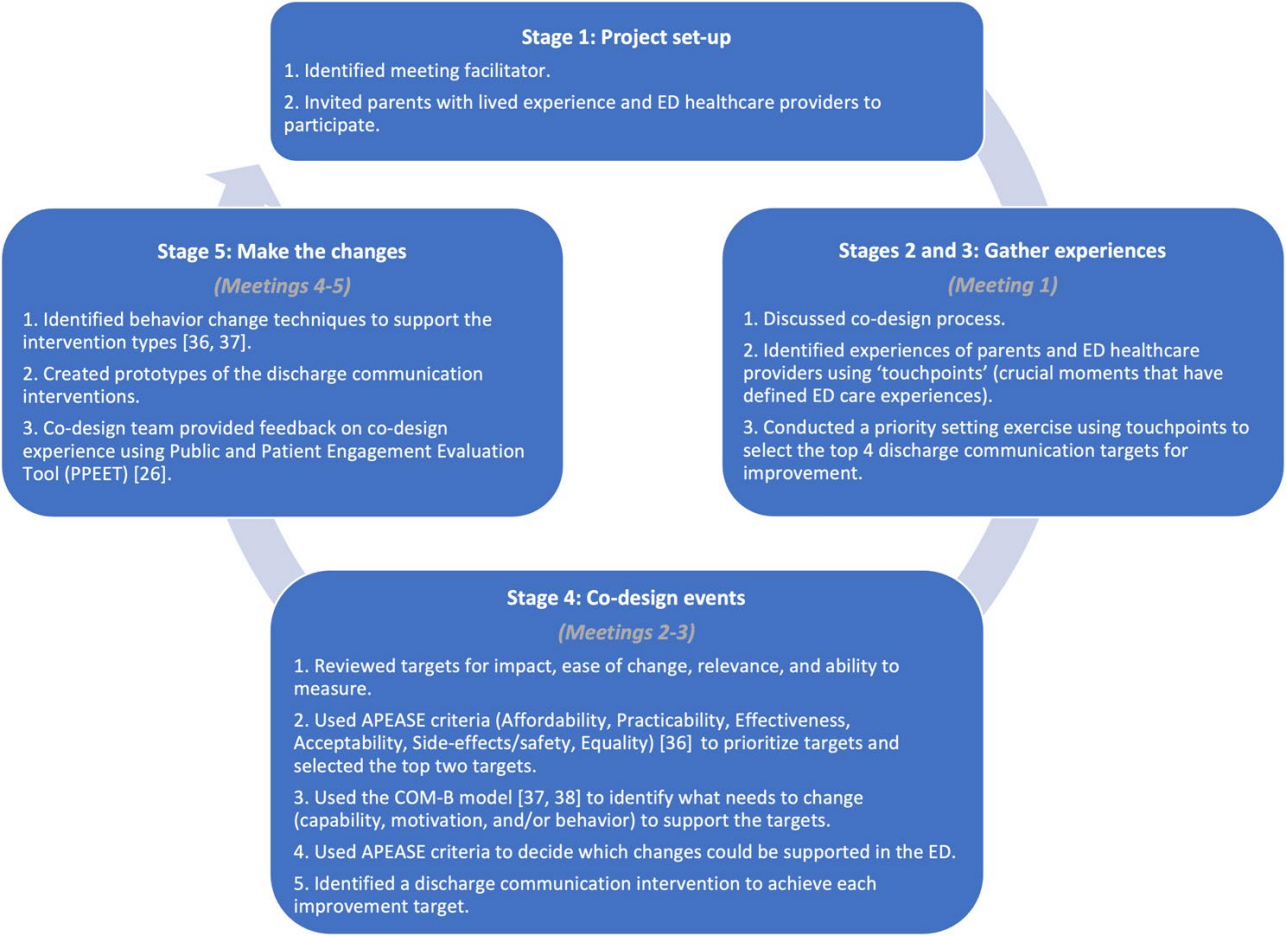
Stages of experience-based co-design incorporated in phase 1

Figure 1 outlines the EBCD process including key actions and decision points for the team. Team members used lived experiences (touchpoints) to identify discharge communication improvement targets, and the APEASE criteria—affordability, practicability, effectiveness, acceptability, side-effects/safety, and equality—to prioritize targets [36]. For the top two ranked targets, the design team identified who needed to be involved in each target, and what behaviors/activities needed to be done and when (and how often). Alongside the APEASE criteria, the team used the Capability, Opportunity, Motivation, Behavior (COM-B) model to identify what needed to change (capability, motivation, and/or behavior) for the target behaviors/activities to occur [36–38] in the Stollery Children’s Hospital ED. The COM-B model outlines that behaviors/activities occur when a person has the capability and opportunity to engage in the behavior, and is motivated to do it. The team discussed whether there was a need for change for each COM-B component and reached a final decision after a consensus vote. Based on this work, prototypes for two discharge communication interventions were developed, and behavior change techniques were identified to support intervention use in clinical practice.

Parents/caregivers evaluated their co-design team experiences by completing the long-term engagement questionnaire from the PPEET [26, 27]. The questionnaire consists of 21 items on processes, outputs, and perceived impacts of engagement activities; 13 questions are scored on a 5-point Likert scale from 1 (strongly disagree) to 5 (strongly agree), and 7 questions are open-ended for comment on scaled items.

### Phase 2 methods

Usability testing was conducted virtually and recorded to facilitate data analysis. Recordings were utilized to create a log that included the most common words used to describe the brochure and usability issues identified by participants. Individual participant sessions took ~ 1-hour to complete and were co-led by two research team members (authors AZA and BW). Parent/caregiver and youth participants completed the medical term recognition test (METER) [39] prior to usability testing to understand the health literacy of those evaluating the interventions.

Sessions were structured according to the think-aloud approach [40], whereby we asked participants to say aloud their thoughts, feelings, and observations as they first viewed the intervention. We also used an interview guide consisting of three open-ended questions (initial impressions, main purpose, usefulness, and/or timing of delivery), one scenario-based question (how the intervention could be used), and asked participants to pick 5–10 words from a list that they felt best described the intervention as they used it. The same approach was used with all participants, but the scenario-based question was tailored to each participant group (parent/caregiver, youth, health care provider). The session concluded with the participant completing a validated user satisfaction survey scoring questions on a 5-point Likert scale from 1 (strongly disagree) to 5 (strongly agree) or 1 (very poor) to 5 (very good) [28]. Health care providers responded to 27 items related to appearance, content, usefulness, and delivery. Parents/caregivers and youth responded to 15 items related to appearance, content, and usefulness. Results from usability testing sessions were presented to the co-design team for intervention refinement.

### Data analysis

We used descriptive statistics to report co-design engagement, participant demographics, and user satisfaction (SPSS, version 23). Meeting minutes collected during co-design meetings were reviewed and coded [41] by one research team member (author AZA) to identify themes within the data related to discharge communication improvement and/or the co-design process. Two research team members (authors AZA and BW) reviewed the coded data together and identified themes. The co-design process themes were used to interpret the PPEET ratings (e.g., instances of decisional conflicts were reviewed to better understand a low PPEET rating). We used the same analytic process [41] to code and thematically group responses to the PPEET open-ended questions and to categorize usability issues identified during the testing sessions.

## Results

### Improvement targets for discharge communication

Three themes for lived experiences with discharge communication were identified by the thematic analysis: (1) confusion about the process of triage and what to do after being discharged, (2) being in shock and forgetting information that was discussed, and (3) not feeling engaged by health care providers in creating a discharge plan for their child. These targets were validated by parents/caregivers and health care provider team members. The co-design team used these experiences to set two improvement targets for discharge communication.

Target one was an interactive discussion between the physician or mental health team member and family before discharge. Its purpose was to ensure engagement when discussing the discharge plan. For this target to be achieved, the co-design team felt that health care providers needed to know the process for engaging families in a conversation about discharge (psychological capability), and see other health care providers engage with families in a discharge conversation (social opportunities). The co-design team also felt that health care providers needed to have dedicated time and resources to engage families in a conversation about discharge (physical opportunities), and have established routines and habits for engaging families in the discharge process (automatic motivation). The team proposed a brochure-based intervention for this target and identified behavior change techniques to support education, enablement, and environmental restructuring, which were considered important for the target to be achieved.

**Fig. 2:**
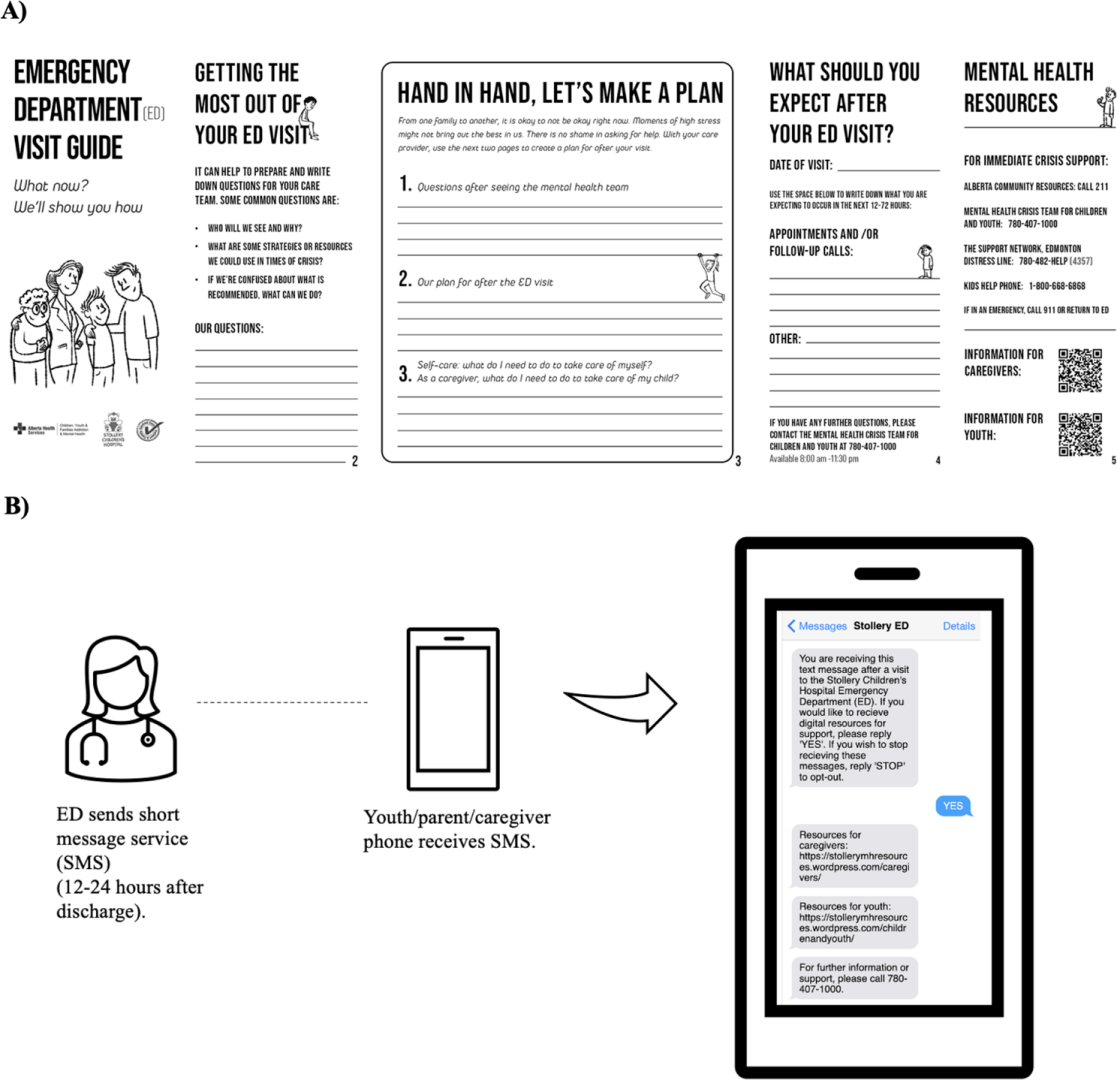
Visual depictions of the two discharge communication interventions. (**A**) Brochure designed to guide engagement during ED visit and creation of discharge plan. (**B**) Text messaging system developed to support families with resources after discharge from ED

Target two was improved communication after the ED visit. For this target to be achieved, the team felt there needed to be a consistent and efficient system to communicate with families after the ED visit (physical opportunity), and established routines for communicating with families after the ED visit (automatic motivation). The team proposed a text message-based intervention for families after ED discharge that would facilitate support, information, and/or guidance depending on the patient/family’s needs. To enhance the uptake of this intervention, the team identified the need for environmental restructuring and enablement, and proposed specific behavior change techniques to support intervention use (Table 2). Additional files provide a detailed overview of the process used by the co-design team to refine details for the two discharge communication targets (see Additional files 2 and 3). Final versions of the brochure and text message-based interventions are visually depicted in Fig. 2.

**Table 2:** An overview of the areas of focus to support behavior change, specific behavior change techniques to facilitate change, and the proposed interventions to help facilitate change

Area of focus	COM-B component addressed by area of focus	Behavior change technique	Proposed discharge communication intervention
**Target behavior 1: Interactive discussion between the physician or mental health team member and family before discharge**			
Education Enablement Environmental restructuring	Psychological capability Physical opportunity Social opportunity Automatic motivation	Add object to the environment	A brochure to be provided to families in the emergency department (ED). *Mode of delivery*: face-to-face
		Prompts/cues	The brochure will contain prompts/cues to help families be engaged during discharge process. ED care providers will be educated on how to use the prompts/cues to engage families during discharge process.
**Target behavior 2: Improve communication after the ED visit**			
Enablement Environmental restructuring	Physical opportunity Automatic motivation	Add object to the environment	Set up a system through which automated text messages can be sent to families after their ED visit. The message will ask if families need further support or resources and connect them with such if needed. *Mode of delivery*: virtual

COM-B: Capability, Opportunity, Motivation, Behavior

### Co-design engagement evaluation

All co-design team members attended at least one of the five co-design meetings; the average number of attended meetings was three. Seven co-design team members (2 health care providers, 5 parents) evaluated their experiences using the PPEET. The results are presented in Table 3. The overall mean score was 4.5/5. The highest rated engagement experiences related to being able to express views freely, feeling heard, and understanding the objectives of the project. The lowest rated engagement experiences related to feeling informed about mental health care through this project, and the range of perspectives being represented. Meeting log notes indicated that clarification was needed regarding the discharge process at the ED (7 instances) and research process for the study (7 instances). Decisional conflicts occurred in 6 instances, where all opinions could not be incorporated into intervention design due to practicality reasons (per APEASE criteria). Themes identified from open-ended questions included participants feeling grateful (opportunity to participate, provide valuable lived experience), learning from other perspectives, and feeling heard. Team members felt that the co-design meetings were conducted in a comfortable and collaborative environment; they suggested having more meeting times available and the opportunity to explore other areas of ED care improvement.

**Table 3:** Score (5-point scale) and response distribution of PPEET items

PPEET item	Mean (SD)
I have a clear understating of the purpose of the discharge communication project.	4.7 (0.5)
The supports I need to participate in the co-design meetings for the discharge communication project are available (e.g., internet access).	4.3 (0.5)
I have enough information to be able to carry out my role.	4.3 (0.5)
I am able to express my views freely.	4.9 (0.3)
I feel that my views are heard.	4.9 (0.3)
A wide range of views on discussion topics is shared.	4.3 (0.7)
The individuals participating in the co-design team for the discharge communication project represent a broad range of perspectives.	4.0 (0.8)
The discharge communication project is achieving its stated objectives.	4.6 (0.5)
I am confident that the feedback provided during our co-design meetings is taken into consideration.	4.6 (0.5)
I think that the work of our co-design meetings makes a difference to the work of the discharge communication project.	4.6 (0.5)
As a result of my participation in the co-design meetings for the discharge communication project, I am better informed about mental health care provided at the Stollery Emergency Department (team members, discharge, and follow-up process).	4.0 (0.5)
Overall, I am satisfied with this engagement initiative.	4.3 (0.5)
This engagement initiative is a good use of my time.	4.6 (0.5)

PPEET: Public and Patient Engagement Evaluation Tool; SD: standard deviation

### Intervention testing

The average participant METER score was 37.3/40 (standard deviation, 4.2), indicating high health literacy among parent/caregiver and youth participants. All usability testing participants identified that the main purpose of the brochure was to help patients and families collaborate on a plan with the care team and provide resources for after discharge. For the brochure, usability themes related to appearance, mental health resources, and instructions for use. The most common words used to describe the brochure were ‘helpful’, ‘useful’, and ‘clear’. Participants reviewed a demonstration of the proposed text message process as the intervention was not yet developed for use. All participants identified that sending a follow-up text with resources or further support would be a helpful, practical way to support families after discharge. Participants did not select words to describe the text messaging system as it could not be used during testing. Themes for anticipated usability issues related to the text messaging system were timing and phrasing of the messages. Issues identified by participants and changes made to the interventions are outlined in Table 4.

**Table 4:** Summary of feedback provided during usability testing cycle

Brochure feature and feedback	Impact on design
**Aesthetics**	
Participants wanted the brochure to be in color, not black and white.	No changes made, as not feasible to print in color at emergency department (ED).
**Resources**	
Provide options for walk-in resources for youth to access.	Added walk-in therapy session information in QR code links.
Include operating hours for mental health crisis team number.	Added information on hours of operation on brochure.
**Instructions**	
Provide instructions (written/verbal) on which sections of brochure to fill out independently vs. with health care provider.	Added written instructions on brochure for sections to be filled out with health care provider.
Provide instructions on which parts of brochure to fill out after being seen by health care provider.	Added written instructions on brochure for section to be filled out after being seen by health care provider.
**Text messaging system feature and feedback**	**Impact on design**
**Timing**	
Send out text message 12–24 h after visit, and during daytime hours.	Text message will be sent out 12–24 h after discharge from ED.
**Phrasing**	
Participants expressed that the phrasing of the text message was misleading, as it implied new resources are being provided.	Text message phrasing was revised to make it clear that digital resources are being provided, if required.

Participants rated their satisfaction with the brochure, but not the text message intervention as it could not be used during testing. Parent/caregiver and youth user satisfaction scores for the brochure ranged from 3 to 5 (mean score, 4.1). The lowest scores were related to appearance, while the highest scores were related to usefulness, understanding, and importance. Health care providers scores ranged from 2 to 5 (mean score, 4.4). Lowest scores related to storing the brochure for occasional use only, and color aesthetics, while highest scores related to brochure understanding, usefulness, and content. Table 5 presents the complete findings.

**Table 5:** Score and response distribution of satisfaction with the brochure

Parent/youth satisfaction	Mean (SD)
At first glance the brochure attracted my attention.	3.0 (0.8)
The brochure held my attention.	3.3 (0.5)
The brochure is useful.	4.8 (0.5)
I like the illustrations on the brochure.	3.0 (1.4)
I believe what the brochure has to say.	3.8 (1.3)
I would recommend the brochure to a friend or relative to use if they presented to the emergency department for a mental health crisis.	4.5 (0.6)
The brochure is easy to understand.	4.5 (0.6)
What the brochure says is important.	4.5 (0.6)
The brochure reminds me of some things I would need to think about if I/ my child presented to the emergency department for a mental health crisis.	4.3 (1.0)
The brochure would give me some new things to think about if I/my child presented to the emergency department for a mental health crisis.	4.5 (0.6)
The brochure changes some of my thinking.	3.3 (0.5)
The brochure could change how I do things.	3.5 (0.6)
Overall, I recommend that emergency department care providers use this brochure in the emergency department with children/youth experiencing a mental health crisis and their families.	5.0 (0.0)
Overall, I am the right person to get this brochure from an emergency department care provider.	4.8 (0.5)
Overall, this brochure accomplishes its main purpose.	4.8 (0.5)
**Health care provider satisfaction**	**Mean (SD)**
**The brochure is designed to**:	
Reinforce information.	4.8 (0.5)
Provide new information.	4.3 (1.0)
Stimulate behavior change.	3.5 (1.9)
At first glance the brochure attracted my attention.	4.3 (0.5)
The brochure held my attention.	4.3 (0.5)
Overall appearance.	4.5 (0.6)
Quality of illustrations.	4.5 (0.6)
Use of color.	3.0 (0.0)
Type face (large enough, attractive, etc.).	4.8 (0.5)
Highlighting of major concepts.	4.8 (0.5)
**The content of the brochure**:	
Up-to-date.	4.8 (0.5)
Scientifically accurate	4.7 (0.6)
Adequate scope for objective(s).	4.5 (0.6)
Overall organization.	4.5 (0.6)
Logical flow of ideas.	4.3 (1.0)
Needed background given to enable understanding.	4.0 (1.0)
Summary(ies) given when needed.	5.0 (0.0)
Fair presentation given (e.g., avoids sexism, ethnic bias, ageism, etc.)	5.0 (0.0)
The brochure is useful for its intended audience.	4.8 (0.5)
The brochure is believable.	4.5 (0.6)
The brochure is understandable.	4.8 (0.5)
The brochure requires little or no explanation.	4.0 (0.8)
Overall, I would recommend that emergency department care providers use this brochure with children/youth presenting with a mental health crisis and their families.	4.8 (0.5)
Overall, this brochure meets its objectives.	4.8 (0.5)
**Brochure placement**:	
The brochure should be given to patients and families in the ED waiting room.	4.8 (0.5)
The brochure should be given to patients and families in the assessment room.	4.0 (1.4)
The brochure should be stored for occasional use.	2.0 (0.8)

## Discussion

Most children and adolescents who come to the ED for a mental health crisis will be discharged home. Discharge interventions should summarize the diagnosis and care given in the ED, address patient questions, teach patients how to care for themselves after the visit, provide information for follow-up care, and may also involve care coordination before leaving the ED [11, 42]. Interventions can be provided verbally or in written or video-based form, or may involve follow-up calls by telephone after the ED visit [43, 44]. However, at this time, comprehensive discharge practices and understandable discharge instructions are lacking for patients and their parents/caregivers, with most discharge interventions being delivered as verbal instructions [11]. In this study, we addressed these clinical care issues by co-designing a brochure to provide written discharge instructions, and a text messaging system to follow up with families after discharge. These interventions can help improve mental health discharge communication practices in the ED and support patient and parent/caregiver recall and understanding of follow-up plans [45, 46].

### The importance of co-design

An important feature of this study was the co-design approach. Traditionally, patients and their parents/caregivers have not been involved in creating new approaches to ED care. This is particularly the case with mental health care [47]. Given that high quality, effective mental health discharge communication requires the involvement of patients, parents/caregivers, and health care providers [48, 49], it was important for us to involve these individuals in intervention development and evaluation. We were mindful of the need to avoid ‘tokenistic engagement’ (e.g., limited influence over defining concerns or solutions) [50] and chose to follow the EBCD framework. EBCD is a best-practice approach to engaging patients in mental health care quality improvement [51] to ensure meaningful engagement throughout the study. This approach can also result in realistic interventions that will be sustainable in clinical practice over time [52].

### Benefits and challenges of using the EBCD framework

The EBCD framework guided us in comprehensively exploring the lived experiences of parents/caregivers through touchpoints and allowed all team members to collectively select target areas for improvement. Having co-design meetings with both parents and clinicians present was extremely beneficial to study progress. There were multiple instances in which clinicians were able to provide feedback to identified changes in discharge communication that parents were interested in. This helped parents/caregivers realize that some identified changes were not feasible or realistic and as such parents/caregivers were able to focus on designing interventions that were realistic, and more likely to be implemented in EDs in the future. The co-design team reported high engagement satisfaction and expressed feeling heard and listened to, further highlighting the benefits of utilizing a framework designed to ensure meaningful engagement. Low engagement ratings and suggested areas for improvement—more flexible meeting times, exploring other ED needs, incorporating more diverse perspectives—are important areas for future projects conducted by our team and others. Some areas for improvement can be readily addressed in future projects such as opening the focus of a project to any area of ED care, not just discharge communication; other areas such as schedule may continue to be a challenge. Despite our best efforts, all co-design team members were not able to attend all meetings due to scheduling conflicts.

### Usability testing

Usability testing was another critical component of this study. Without this method, we may not have identified issues with acceptability, usability, or identified issues that can be used as part of an implementation strategy to support routine intervention use [53]. Conducting formal usability testing revealed that the interventions were helpful, clear, and useful to the desired population and was critical to identify important areas for improvement in the designed interventions. The feedback from the usability testing helped us adapt the interventions to be more user-friendly and supportive by including more detailed instructions and further mental health resources.

We believe that the interventions developed in this study can support discharge communication for a mental health visit. The brochure aims to guide the conversation between pediatric patients, parents/caregivers, and ED health care providers, and provides a place to document, during their visit, important concerns and treatment and follow-up plans. However, as the brochure has not yet been implemented in an ED setting, further evaluation will be needed to test the impact of the brochure on patient and family outcomes and experiences, such as the comprehension and recall of discharge plans and satisfaction with care received. The text messaging intervention aims to support families in the post-crisis period. Questions or concerns that emerge after the ED visit, or the need to clarify discharge instructions, can also be addressed through this intervention. This intervention is similar to Caring Contacts, a suicide prevention approach that involves sending brief messages to patients after discharge to provide resource information and support [54, 55]. Because the text messaging system has yet to be developed, additional usability testing is required once the system is ready for use. Developing the text messaging system will involve making the texts more personalized if possible. Testing should include rating experiences with the System Usability Scale (SUS) and/or Severity Ranking Scale (SRS) to understand the usability of this technology [56]. The SUS has published cut-points for interpreting usability (acceptable, not acceptable) and the SRS serves to rate concerns with any features of the technology (none, cosmetic, minor, major, catastrophic). Future studies are also needed to test the impacts of both new interventions on patient and family outcomes and experiences (e.g., anxiety, stress, care satisfaction), as well as the impact on health care system use after the ED visit (e.g., follow-up visit rates, ED re-visits) to understand the potential value of these new interventions.

### Understanding behavior change

Our use of an evidence-based, behavior change framework in this study allowed us to address all aspects that affect change (motivation, capability, and opportunity) as well as establish, recognize, and describe the pathways or mechanisms underpinning the discharge communication interventions [36]. While we have yet to test the impact of the two new interventions, our approach to intervention development will allow us to test not only the effects of the discharge intervention on patient care and outcomes (intervention effectiveness), but also the effects of strategies used to support intervention use (implementation strategy effectiveness) [57]. Hybrid effectiveness-implementation studies for pediatric care have been conducted in ED settings [58, 59], providing important information on both intervention impacts and how to optimize intervention use.

## Limitations

There are several limitations to this study. First, the targets for change and design of the discharge communication interventions were conceptualized from a small sample of parents and health care providers from one children’s hospital, which may limit applicability of study findings to other ED settings. Second, we were unable to recruit adolescents or youth to the co-design team, which would have allowed us to incorporate the patient perspective into intervention design. Third, although the co-design process was collaborative, team members could not attend all meetings due to scheduling conflicts, which limited consistent involvement. Fourth, our project scope was limited to testing the co-design process and to test for usability – we were not aiming to study the impact of the discharge communication interventions on health outcomes.

## Conclusions

The interventions produced by this study have the potential to address gaps in current discharge practices. Our study included several important frameworks and methods—EBCD, usability testing, and behavior change—to design and initially test two, novel discharge communication interventions. This approach resulted in the development of interventions that reflect the needs and preferences of health care users/deliverers. Future testing is required to evaluate the impact on patients, caregivers, and health care system after the ED visit.

### Electronic supplementary material

Below is the link to the electronic supplementary material.

Supplementary Material 1

Supplementary Material 2

Supplementary Material 3

## Data Availability

All data generated or analysed during this study are included in this published article [and its supplementary information files].

## Abbreviations

ED: Emergency Department
EBCD: Experience-based Co-design
GRIPP: Guidance for Reporting Involvement of Patients and the Public
APEASE: Affordability, Practicability, Effectiveness, Acceptability, Side-effects/safety, and Equality
LGBTQ: Lesbian, Gay, Bisexual, Transgender, Queer
COM-B: Capability, Opportunity, Motivation, Behavior
PPEET: Public and Patient Engagement Evaluation Tool
METER: Medical Term Recognition Test
SD: Standard Deviation
SUS: System Usability Scale
SRS: Severity Ranking Scale
