# Supplementary material for: Co-designing discharge communication interventions for mental health visits to the pediatric emergency department: a mixed-methods study
Source: Res Involv Engagem. 2024 Jun 21;10:64. doi: 10.1186/s40900-024-00594-y (PMC11191193; doi:10.1186/s40900-024-00594-y)
Supplement: Supplementary file 2 — Supplementary Material 2 [file 40900_2024_594_MOESM2_ESM.docx]

**Additional File 2**. Behavioral analysis of improvement targets using the COM-B model.

| COM-B component | What needs to happen for the target to take place? | Is there a need for change? |
| --- | --- | --- |
| *Target behavior 1: Interactive discussion between the physician or mental health team member and family before discharge* | | |
| Physical capability | Have the physical skills to engage families in a conversation about discharge. | No change needed as care providers have these skills. |
| Psychological capability | Know the correct process of engaging families in a conversation about discharge. | Change needed as sometimes nurses do not know if patients are ready for discharge and are therefore hesitant to engage families in a discharge conversation. |
| Physical opportunity | Have the dedicated time and resources to engage families in a conversation about discharge. | Change needed as there are no resources available to ensure families are engaged, and a lack of time to develop relationships with patients and families. |
| Social opportunity | See other health care providers engage families in a conversation about discharge. | Change needed as most care providers do not do this consistently. |
| Reflective motivation | Hold beliefs that engaging families is important to discharge process. | No change needed as care providers do hold these beliefs. |
| Automatic motivation | Have established routines and habits for engaging families in discharge process. | Change needed to establish routine and habit formation. |
| *Target behavior 2: Improve communication after the ED visit* | | |
| Physical capability | Have the physical skills to communicate with families after the ED visit. | No change needed as care providers have these skills. |
| Psychological capability | Know the correct procedure for communicating with families after the ED visit. | No change needed as care providers know the current procedure and are capable of learning a new procedure. |
| Physical opportunity | Have a consistent and efficient system set-up to communicate with families after ED visit. | Change needed as there is currently no consistent system set-up. |
| Social opportunity | See other care providers or staff reaching out to families after ED visit. | No change needed as care providers/staff already see this. |
| Reflective motivation | Hold beliefs that communication with families is important after an ED visit. | No change needed as care providers hold these beliefs. |
| Automatic motivation | Have established routines for communicating with families after the ED visit. | Change needed to establish routine and habit formation. |

COM-B: Capability, Opportunity, Motivation, Behavior
