# Supplementary material for: Co-designing discharge communication interventions for mental health visits to the pediatric emergency department: a mixed-methods study
Source: Res Involv Engagem. 2024 Jun 21;10:64. doi: 10.1186/s40900-024-00594-y (PMC11191193; doi:10.1186/s40900-024-00594-y)
Supplement: Supplementary file 3 — Supplementary Material 3 [file 40900_2024_594_MOESM3_ESM.docx]

**Additional File 3**. Use of the COM-B model and APEASE criteria to refine details for the two discharge communication targets.

| Target | Potential strategy to support behavior change (COM-B model) | Does the potential strategy meet APEASE criteria? |
| --- | --- | --- |
| Target 1: Interactive discussion between the ED physician or mental health team member and family. | Education | Yes |
|  | Training | Not practicable; not enough time or resources to train all staff. |
|  | Enablement | Yes |
|  | Restriction | Not practicable; no options to restrict in this context. |
|  | Environmental restructuring | Yes |
|  | Persuasion | Unlikely to be effective in an ED setting. |
|  | Incentivization | Not practicable in an ED setting. |
|  | Coercion | Not practicable in an ED setting. |
|  | Modelling | Not practicable in an ED setting. |
| Target 2: Improve communication after the ED visit. | Persuasion | Unlikely to be effective without resources and time. |
|  | Incentivization | Not practicable in an ED setting. |
|  | Coercion | Not practicable in an ED setting. |
|  | Training | Unlikely to be effective without a system set-up to communicate through. |
|  | Environmental restructuring | Yes |
|  | Modelling | Not practicable in an ED setting. |
|  | Enablement | Yes |
|  | Restriction | Not practicable; no options to restrict in this context. |

APEASE: affordability, practicability, effectiveness, acceptability, side-effects/safety, and equality

COM-B: Capability, Opportunity, Motivation, Behavior
